# Novel rare variations in genes that regulate developmental change in N-methyl-d-aspartate receptor in patients with schizophrenia

**DOI:** 10.1038/hgv.2017.56

**Published:** 2018-02-01

**Authors:** Akane Yoshikawa, Fumichika Nishimura, Aya Inai, Yosuke Eriguchi, Masaki Nishioka, Atsuhiko Takaya, Mamoru Tochigi, Yoshiya Kawamura, Tadashi Umekage, Kayoko Kato, Tsukasa Sasaki, Kiyoto Kasai, Chihiro Kakiuchi

**Affiliations:** 1 Department of Neuropsychiatry, Graduate School of Medicine, The University of Tokyo, Tokyo, Japan; 2 Department of Child Neuropsychiatry, Graduate School of Medicine, The University of Tokyo, Tokyo, Japan; 3 Division for Counseling and Support, Office for Mental Health Support, The University of Tokyo, Tokyo, Japan; 4 Department of Psychiatry, Fukui Memorial Hospital, Kanagawa, Japan; 5 Department of Neuropsychiatry, Teikyo University School of Medicine, Tokyo, Japan; 6 Department of Psychiatry, Shonan Kamakura General Hospital, Kamakura, Japan; 7 Division for Environment, Health and Safety, University of Tokyo, Tokyo, Japan; 8 Department of Health Education, Graduate School of Education, The University of Tokyo, Tokyo, Japan; 9 Disability Services Office, The University of Tokyo, Tokyo, Japan

## Abstract

The mechanism underlying the vulnerability to developing schizophrenia (SCZ) during adolescence remains elusive. Hypofunction of *N*-methyl-d-aspartate receptors (NMDARs) has been implicated in the pathophysiology of SCZ. During development, the composition of synaptic NMDARs dramatically changes from NR2B-containing NMDARs to NR2A-containing NMDARs through the phosphorylation of NR2B S1480 or Y1472 by CDK5, CSNK2A1, and EphB2, which plays a pivotal role in the maturation of neural circuits. We hypothesized that the dysregulation of developmental change in NMDARs could be involved in the onset of SCZ. Using next-generation sequencing, we re-sequenced all the coding regions and splice sites of *CDK5*, *CSNK2A1*, and *EphB2* in 474 patients with SCZ and 475 healthy controls. Variants on the database for human control subjects of Japanese origin were removed and all the nonsynonymous and nonsense variants were validated using Sanger sequencing. Four novel variants in *CDK5* were observed in patients with SCZ but were not observed in controls. The total number of variants, however, was not significantly different between the SCZ and control groups (*P*=0.062). In silico analyses predicted P271T to be damaging. Further genetic research using a larger sample is required to examine whether *CDK5* is involved in the pathophysiology of SCZ.

## Introduction

The onset of schizophrenia (SCZ) usually occurs during early adolescence. SCZ is a neurodevelopmental disorder with a strong genetic component and is highly related to disturbances in brain/circuit maturation.^1–3^ However, the molecular mechanisms underlying the vulnerability for SCZ during early adolescence remain elusive.

Accumulating evidence has demonstrated that hypofunction of N-methyl-d-aspartate receptors (NMDARs) is involved in the pathophysiology of SCZ.^4–11^ This hypothesis was initially based on clinical findings that the uncompetitive NMDAR antagonists, phencyclidine (PCP) and ketamine, induced psychotic features resembling symptoms seen in patients with SCZ. These features included both positive and negative symptoms, as well as cognitive dysfunction in healthy volunteers.^5^ In addition, PCP exacerbated these symptoms in patients with chronic SCZ.^12^ Further, *GRIN2A*, which encodes the NR2A subunit of NMDARs, has been consistently reported to be associated with SCZ ^9–11^ and has reached genome-wide significance in the largest genome-wide association study (GWAS) performed by the Psychiatric Genomics Consortium.^13^

A large exome-sequencing study using 617 trios also revealed that patients with sporadic SCZ harbored *de novo* mutations in genes related to NMDARs and in the activity-regulated cytoskeleton-associated protein (ARC) complex, including *GRIN2A* and *DLG.*^14^ Purcell *et al.* showed additional evidence of NMDAR hypofunction by identifying rare, disruptive mutations in NMDAR and ARC gene sets through exome-sequencing of 2536 patients with SCZ.^15^

NMDARs play a crucial role in synaptic plasticity, neuronal development, learning, and memory.^16,17^ Each NMDAR is a tetramer composed of two NR1 subunits and two NR2 or NR3 subunits.^18^ The number, localization and subunit composition of NMDARs are strictly regulated in an age-dependent manner so that they can exert their function precisely.^16,18^ The NR2 subtype, that is, NR2A-NR2D, defines the pharmacological and biophysical properties of the NMDAR.^16,19,20^ NR2A-containing NMDARs are considered to be more neuroprotective than NR2B-containing NMDARs depending on their Ca^2+^ permeability.^21,22^

During development, the composition of NMDARs dramatically changes from those that contain NR2B subunits to those that contain NR2A.^23,24^ This NMDAR subunit switch (NMDAR switch) plays an important role in the maturation of neural circuits, especially in the GABAergic inhibitory circuit in the prefrontal cortex, in a synaptic activity-dependent manner.^16,18,25^

Recent evidence also revealed that the NMDAR subunit switch is mediated by the endocytosis of NR2B, which is induced by phosphorylation of NR2B S1480 or Y1472 during early development.^26^ These phenomena are induced by cyclin-dependent kinase 5 (CDK5), casein kinase 2 (CK2), and erythropoietin-producing hepatocellular carcinoma group B (EphB2), whose activities are also regulated in an age-dependent manner.^26–30^

The effect of PCP on NMDARs differs according to the developmental stage and depends on the NR2 subunit composition.^31,32^ Postmortem brain studies in SCZ patients have shown a reduction in the mRNA expression of *GRIN2A*, which encodes NR2A, on parvalbumin-positive interneurons in the prefrontal cortex, and an increase in the mRNA expression of *GRIN2B*, which encodes NR2B, in the dorsomedial thalamus, temporal cortex, and hippocampus.^6,33–35^ A decrease in CK2 expression and dysregulation of CDK5-P35/P25 has been reported.^36–38^ CDK5 has been shown to phosphorylate the dopamine D2 receptor, which is involved in the pathophysiology of SCZ.^39^ In addition, 7 microRNAs that commonly regulate the expression of CDK5 were reported to be potential peripheral biomarkers for SCZ.^40^ Aripiprazole increased the ratio of NR2A/NR2B in rats, while risperidone increased the activity of *CK2*.^38,41^ Furthermore, EphB2 activates kalirin-7, leading to activity-dependent synaptic formation; knockdown mice for this receptor showed SCZ-like behavioral abnormalities after the period of adolescence.^42^

GWAS have successfully provided evidence that the common variants in genes related to synaptic function contribute to disease risk in SCZ. However, the problem of missing heritability has emerged because only 23% of the heritability can be explained by current GWAS findings.^43^ Identifying rare variants with a strong effect is another alternative strategy to reveal the genetic architecture of SCZ.^43,44^

In the current study, we hypothesized that the dysfunction of adolescence-related neurodevelopmental events, especially the NMDAR subunit switch, which is regulated by phosphorylation of NR2B, S1480, or Y1472, might contribute to the onset of SCZ. Thus, we re-sequenced *CDK5* (**BC005115**), *CSNK2A1* (**BC050036**), and *EphB2* (**BC067861**) to examine the genetic contribution of these genes, focusing on rare variants to examine the pathophysiology of SCZ.

## Materials and methods

### Participants

A total of 474 patients with SCZ (234 males and 240 females, age 43.12±15.14 years) and 475 control subjects (224 males and 251 females, age 41.54±12.43 years) who were unrelated were analyzed in the current study. There were no significant differences in sex and age between the two groups (Student’s *t*-test). Inpatients and outpatients of the Department of Neuropsychiatry, University of Tokyo Hospital, and related hospitals and daycare facilities located around Tokyo were recruited for this study. Two or more senior psychiatrists diagnosed the patients through interviews and clinical records using the Diagnostic and Statistical Manual of Mental Disorders DSM-IV criteria (American Psychiatric Association). Patients with a history of drug addiction and alcohol abuse/dependence were excluded. Using the Mini-International Neuropsychiatric Interview^45^ or unstructured interviews, control subjects with current or lifetime mental illness, alcohol abuse/dependence, or a family history of any psychiatric disorder were also excluded. All participants were ethnically Japanese. The study was performed in accordance with the Declaration of Helsinki. The Research Ethics Committee of the Faculty of Medicine, University of Tokyo approved this study (Approval No. G0639-(33)). Written informed consent was obtained from all participants after they were provided an explanation of the study.

### Re-sequencing

Genomic DNA was isolated from leukocytes derived from whole blood using a DNA blood kit (QIAGEN Ltd., Hilden, Germany), as previously described.^46^ A total of 250 ng of genomic double-stranded DNA was used in the target re-sequencing experiment. Target re-sequencing of all the coding regions and the splice sites (±5 base pairs (bp)) of *CDK5*, *CSNK2A1*, and *EphB2* was performed to identify missense/nonsense variants and small insertions/deletions using a uniquely customized design TruSeq Custom Amplicon (Illumina, San Diego, CA, USA) with MiSeq (Illumina, California, USA) according to the manufacturer’s instructions.^47^ Human *CDK5*, *CSNK2A1*, and *EphB2* are located on chromosomes 7q36, 20q13, and 1p36.12, respectively. Briefly, 250 ng of total double-stranded DNA was used. Amplicons were designed using Design Studio (http://designstudio.illumina.com/), and 59 amplicons were available. After hybridization, ligation, and amplification, the samples were applied to the sequencer. Raw FASTQ files from the sequencer were retrieved, and short reads were aligned to the hg19 reference using the Burrows-Wheeler Aligner software (http://bio-bwa.sourceforge.net/).^48^ Picard tools were used to remove duplicates. Subsequently, SAMtools (http://samtools.sourceforge.net/) was used to convert to BAM files and for variant calling.^49^ GATK was used for base quality score recalibration and was also used to detect SNV and small indels. VCF files were ultimately obtained, and variants were extracted. Using IGV, we visualized the next-generation sequencing data. Sanger sequencing was performed to sequence the genomic region when no amplicon was available (*CDK5* exon 1 (Chr7: 150754894–150754935), exon 4 (Chr7: 150753818–150753888), exon 5 (Chr7: 150753662–150753728), *CSNK2A1* exon 4 (Chr20: 485757–485878), *EphB2* exon 1 (Chr1: 23037476–23037541)) and was used to validate all genetic variants using a 3130XL Genetic Analyzer (Applied Biosystems, Foster City, CA, USA) after removal of the variants detected in a healthy database cohort of Japanese origin (Human Genetic Variation Database: http://www.genome.med.kyoto-u.ac.jp/SnpDB/index.html; HGVD). The primers were designed using Primer 3 software (http://bioinfo.ut.ee/primer3-0.4.0/primer3/). Primer pairs used in Sanger sequencing are shown in Supplementary Table S1.

### *In silico* analysis

We predicted the functional effect of the variants that were detected using the database for nonsynonymous single nucleotide polymorphisms’ functional predictions (dbNSFP; https://sites.google.com/site/jpopgen/dbNSFP), an integrated functional analysis database of SIFT, Polyphen2, LRT, Mutation Taster, and FATHMM.^50^

### Statistical analysis

Student’s *t*-test was used to compare the ages and sexes of participants in the SCZ and control groups. Fisher’s exact test was used to examine whether the number of the variants not reported in the HGVD was significantly overrepresented in patients with SCZ compared with controls.

## Results

### Variants observed in *CDK5*, *CSNK2A1*, and *EphB2*

**Figure 1:**
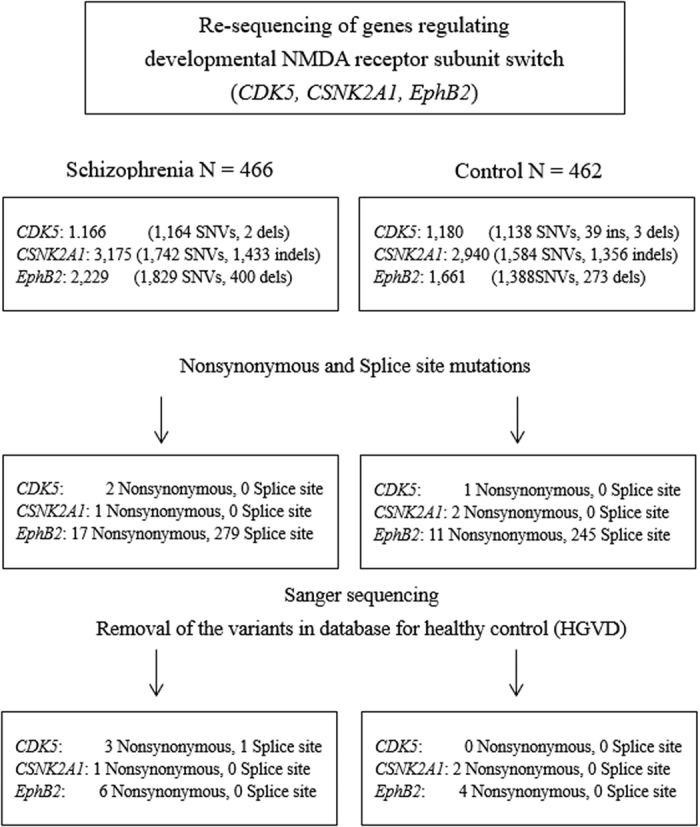
Flow diagram of processes used to detect single nucleotide variations (SNVs) and insertions and deletions (indels) in N-methyl-d-aspartate (NMDA) receptor switching regulator genes in patients with schizophrenia (SCZ) and in controls. Target re-sequencing of entire coding regions and splice sites (±5 base pairs) were performed. All detected nonsynonymous variants and regions with low coverage (<20), with no available designed amplicons were examined by Sanger sequencing. After sample quality control, 8 patients and 13 controls were excluded due to low DNA quality. Three nonsynonymous and one splice region variant in *CDK5* were observed in patients with SCZ but not in controls. Nonsynonymous variants of *CSNK2A1* and *EphB2* were identified in both patients with SCZ and controls.

We performed target re-sequencing of *CDK5*, *CSNK2A1,* and *EphB2,* genes that are shown to regulate the NMDAR switch via phosphorylation of NR2B Y1472 or S1480, in 474 patients with SCZ and 475 controls. The flowchart of the analysis is shown in Figure 1. A total of 8 patients and 13 controls were excluded due to low DNA quality. All of the variants that were identified (Table 1) were novel single-base heterozygous substitutions.

**Figure 2:**
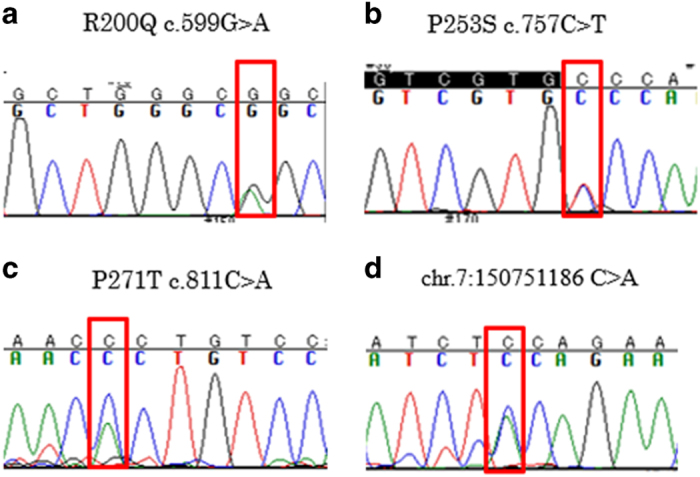
The electropherogram traces of variants confirmed by Sanger sequencing in *CDK5*. The traces include four novel variants located in the exons of *CDK5* (**a**–**c**) and one variant in the splice region (**d**). The locations, nucleotide changes, and consequent amino acid changes are described in the subfigures.

In *CDK5*, three nonsynonymous variants (i.e., R200Q in exon 9, P253S in exon 11, P271T in exon 12) and one splice region variant (i.e., Chr7:150751186, C to A) were observed in patients with SCZ but not in controls (Figures 2a–d).

In *CNSK2A1,* one nonsynonymous variant (M339I) in exon 13 was observed in patients with SCZ, and two nonsynonymous variants (M339I in exon 13 and R134Q in exon 7) were observed in controls after removing the variants that were observed in the healthy cohort database.

In *EphB2,* we observed five nonsynonymous variants (R80H in exon 3, R229S in exon 3, V268I in exon 3, R569W in exon 9, and G734V in exon 12) and four nonsynonymous variants (N133S in exon 3, V190M in exon 3, V465M in exon 6, and R569M in exon 9) in six patients with SCZ and four controls. R569W was observed in a patient with SCZ and in a control.

The total number of variants in each gene was not significantly different between the two groups (*CDK5*: *P*=0.062, *CSNK2A1*: *P*=0.624, *EphB2*: *P*=1.000).

### *In silico* analysis

Next, we performed a functional prediction of the variants we observed in this study using dbNSFP. In this analysis, P271T in *CDK5* in patients with SCZ was predicted to be highly damaging (Table 1). Furthermore, M339I, R134Q in *CSNK2A1*, G734V, and V190M in *EphB2* were also predicted to be highly damaging.

## Discussion

To examine whether the developmental NMDAR subunit switch was genetically involved in the pathophysiology of SCZ, we performed target re-sequencing of *CDK5*, *CSNK2A1*, and *EphB2*, that regulate developmental NMDAR subunit switch via phosphorylation of NR2B Y1472 or S1480, using samples from 474 patients with SCZ and 475 controls. This is the first study focusing on the genetic relationship between the developmental change in NMDAR subunit switching and SCZ, although we failed to provide evidence for an NMDAR switch in the current study.

In *CDK5*, we observed four novel variants in patients with SCZ but not in controls, although the total number of variants detected was not significantly different between the two groups (*P*=0.062). The crystal structure of the cAMP-dependent protein kinase, including CDK5, has been shown to have an N-terminal domain (N lobe) that is composed of mainly ß-sheets, a helical C-terminal domain (C lobe), and a deep ATP-binding cleft between the two lobes.^51^ According to InterPro (https://www.ebi.ac.uk/interpro/), a database for predicting protein function, P271 is located at the helical C-terminus in *CDK5* and belongs to the protein kinase domain that is crucial for its serine/threonine protein kinase activity. The tyrosine (Y1472) within the YEKL motif of NR2B was shown to be phosphorylated by the CDK5, serine/threonine protein kinase. Phosphorylation of this site inhibited the binding of AP-2 to YEKL motif, which is necessary for NR2B endocytosis, and promoted synaptic surface expression of NR2B-containing NMDARs instead of reducing the surface expression of NR2A-containing NMDARs.^27^ These findings raise the possibility that the P271T found in this study may affect the regulation of the developmental NMDARs switch. Another possibility is that P271T may disrupt the function of CDK5 by interrupting the formation of the CDK5–p25 complex, as the α-helix is a critical structure in the interaction between CDK5 and p25, a co-activator of CDK5.^51–53^ Furthermore, the C-terminus of the helix is a well-conserved region among spices^51,54^ and may lead to a highly damaging effect on its function.

In a previous large exome study of 2536 patients with SCZ and 2543 controls (http://research.mssm.edu/statgen/sweden/),^15^ a rare, nonsynonymous variant, R50W, in *CDK5* was detected in patients with SCZ but not in controls. In the subsequent updated exome study of 4877 patients with SCZ and 6203 controls showed that there was one control subject with p. P170L (c.509 C>T) in *CDK5* but no missense or nonsense variants in patients with SCZ. R50W was later found in the largest exome database, the Exome Aggregation Consortium. We further combined the data from the largest SCZ exome study and our study to examine whether the number of rare variants in *CDK5* was significantly different between SCZ patients and controls, as both studies referred to rare variants with MAF<0.5%. The total number of rare variants within *CDK5* was not statistically significant (*P*=0.179, Fisher’s exact test). Additional research using a larger sample size may be required, as the races and pipeline for detecting the rare variants were different between the studies.

Recently, excessive dopamine D2 receptor activation during early adolescence was shown to prevent the development of dendritic spines and led to cognitive dysfunction, a core symptom of SCZ, in an NR2B-dependent manner.^55^ Thus, the dysregulation of the composition of NMDARs may affect the dopamine levels during early adolescence, thereby affecting synapse maturation.^56^ Therefore, the involvement of other novel switching regulator genes warrants further research.

In conclusion, we examined whether the developmental NMDAR subunit switch was genetically involved in the pathophysiology of SCZ by re-sequencing three genes that regulate the composition of NMDARs. We found four novel variants in *CDK5* in patients with SCZ but not in controls, although the difference did not show statistical significance. Additional research using a larger sample size or examining other switching regulator genes is warranted.

## Additional infomation

**Publisher’s note:** Springer Nature remains neutral with regard to jurisdictional claims in published maps and institutional affiliations.

## Figures and Tables

**Table 1:** Variations in the NMDA receptor switch regulator genes and the functional prediction analysis in patients with schizophrenia and in controls

*Gene*	*ID*	*Chr*	*Location*	*Ref*	*Alt*	*Variants*	*Total dbNSFP score*	*SIFT*^a^		*Polyphen2*		*LRT*^a^		*Mutation Taster*		*FATHMM*^a^	
*CSNK2A1*	SZ-01	chr20	467063	C	T	M339I	4.212^b^	1.000	T	1.000	D	1.000	D	1.000	D	0.212	T
*CSNK2A1*	CT-01	chr20	467063	C	T	M339I	4.212^b^	1.000	T	1.000	D	1.000	D	1.000	D	0.212	T
*CSNK2A1*	CT-02	chr20	478390	C	T	R134Q	4.188^b^	0.910	T	1.000	D	1.000	D	1.000	D	0.278	T
*EphB2*	SZ-21	chr1	23110997	G	A	R80H	4.261^b^	1.000	D	1.000	D	1.000	D	1.000	D	0.261	T
*EphB2*	SZ-22	chr1	23111444	A	G	N229S	3.174	0.690	T	0.282	B	1.000	D	1.000	D	0.202	T
*EphB2*	SZ-23	chr1	23111560	G	A	V268I	3.139	0.700	T	0.164	B	0.993	N	0.979	N	0.303	T
*EphB2*	SZ-24	chr1	23222908	C	T	R569W	3.286	0.970	D	0.008	B	1.000	D	1.000	D	0.308	T
*EphB2*	SZ-25	chr1	23222908	C	T	R569W	3.286	0.970	D	0.008	B	1.000	D	1.000	D	0.308	T
*EphB2*	SZ-26	chr1	23234507	G	T	G734V	4.417^b^	0.990	D	1.000	D	1.000	D	1.000	D	0.427	T
*EphB2*	CT-21	chr1	23111156	A	G	N133S	2.971	0.610	T	0.008	B	1.000	D	0.995	D	0.287	T
*EphB2*	CT-22	chr1	23111326	G	A	V190M	4.280^b^	1.000	D	1.000	D	1.000	D	1.000	D	0.280	T
*EphB2*	CT-23	chr1	23208941	G	A	V465M	3.975	0.980	D	0.613	PD	1.000	D	0.999	D	0.383	T
*EphB2*	CT-24	chr1	23222908	C	T	R569W	3.286	0.970	D	0.008	B	1.000	D	1.000	D	0.308	T
*CDK5*	SZ-31	chr7	150752165	C	T	R200Q	3.335	0.930	T	0.095	B	1.000	D	1.000	D	0.405	T
*CDK5*	SZ-32	chr7	150751334	G	A	P253S	3.391	0.970	D	0.058	B	1.000	D	1.000	D	0.363	T
*CDK5*	SZ-33	chr7	150751164	C	A	P271T	4.371^b^	0.990	D	1.000	D	1.000	D	1.000	D	0.381	T
*CDK5*	SZ-34	chr7	150751186	C	A	Splice 5′+4	N/A	N/A		N/A		N/A		N/A		N/A	

Abbreviations: Alt alteration, Chr chromosome, CT controls, dbNSFP database for nonsynonymous SNPs' functional predictions, D damaging, N neutral, N/A not available, PD possibly damaging, Ref reference allele, SZ schizophrenia, Splice Splice region variant, T tolerant.

a converted.

b dbNSFP score total: >4, deleterious.
