# Supplementary material for: Novel rare variations in genes that regulate developmental change in N-methyl-d-aspartate receptor in patients with schizophrenia
Source: Hum Genome Var. 2018 Feb 1;5:17056–. doi: 10.1038/hgv.2017.56 (PMC5794673; doi:10.1038/hgv.2017.56)
Supplement: Supplementary Table S1 [file hgv201756-s1.docx]

**Supplementary table S1. Primer pairs used in this study**

Primers Product size Sequences

CSNK2A1-exon4F 342bp 5’-aaaacctatttgggaattgtttga -3’

CSNK2A1-exon4R 342bp 5’-gctttggcaccaaaaacctt -3’

CSNK2A1-exon6F 248bp 5’-caacggtaaatgagccaagtct -3’

CSNK2A1-exon6R 248bp 5’-atctgctggcttttctaccatc -3’

CSNK2A1-exon7F 259bp 5’-tcaggcttccagaccattagtt -3’

CSNK2A1-exon7R 259bp 5’-caccagtacggagaagagaagg -3’

CSNK2A1-exon8F 299bp 5’-tgactgtgtcctcagcttgaat -3’

CSNK2A1-exon8R 299bp 5’-cctgtgacaacacaagggataa -3’

EphB2-exon1F 427bp 5’-tggatggctcattctgct -3’

EphB2-exon1R 427bp 5’-cctggcacagtcaatcagc -3’

EphB2-exon2F 253bp 5’-aagaaggatgagggcccaac -3’

EphB2-exon2R 253bp 5’-gtcctgaagagggggaaagg -3’

EphB2-exon3F 269bp 5’-tcaccattagactggagggttc -3’

EphB2-exon3R 269bp 5’-caccgaaaacttcatctccac -3’

EphB2-exon4F 297bp 5’-gctcgtgacctctctgagtctt -3’

EphB2-exon4R 297bp 5’-ccaatcctcactctgagctttt -3’

EphB2-exon5F 484bp 5’-gagtggtcacatggggaatagt -3’

EphB2-exon5R 484bp 5’-aggagtcttttgtgaggactc -3’

EphB2-exon6F 342bp 5’-cttccctctctgatcccacagt -3’

EphB2-exon6R 342bp 5’-caccagtgcgtctcatcttg -3’

EphB2-exon9F 242bp 5’-ccaccctctccctatcacctac -3’

EphB2-exon9R 242bp 5’-atggcagaaggaggtcaaagat -3’

EphB2-exon12F 385bp 5’-cattatgaggatgatgcagagc -3’

EphB2-exon12R 385bp 5’-aattgggcgttagtgaaagtgt -3’

EphB2-exon14F 384bp 5’-caatgtactgggggtaagatgg -3’

EphB2-exon14R 384bp 5’-gctgcagtaaggaagagcattt -3’

EphB2-exon15F 369bp 5’-tcacataaatggatgcacacg -3’

EphB2-exon15R 369bp 5’-atgatctattcctcaccctcca -3’

EphB2-exon16F 297bp 5’-cttgctttgccatcttcctc -3’

EphB2-exon16R 297bp 5’-cagtggatagagcaccaggag-3’

CDK5-exon1F 250bp 5’-tttaggactacaagccccagaa -3’

CDK5-exon1R 250bp 5’- tggaggctcacacagttagga-3’

CDK5-exon2F 254bp 5’-atttcctggcttagggaagagt -3’

CDK5-exon2R 254bp 5’-ggcacaccctgcatatgtga -3’

CDK5-exon6F 299bp 5’-gaagcacaagggtgaggagag -3’

CDK5-exon6R 299bp 5’-cctttaaaaagccttgggacag -3’

CDK5-exon7F 262bp 5’- gagcacagagggaagaggact-3’

CDK5-exon7R 262 bp 5’-ggctcagagaggagaggaaaat -3’

CDK5-exon8F 271bp 5’-cccaggacccaaaacattatt -3’

CDK5-exon8R 250bp 5’-gaccctccagaccctatttgtc -3’

CDK5-exon10F 290bp 5’-gtaaagggagggtgagaagtgg -3’

CDK5-exon10R 290bp 5’-cagggagggtcagactagaggt -3’

CDK5-exon11F 256 bp 5’-atcagggtcactcgtttcacat -3’

CDK5-exon11R 256bp 5’-gcaggttcaggacaggtcac -3’

CDK5-exon12F 353bp 5’-aggtaggtgaccaggggtagag -3’

CDK5-exon12R 353bp 5’-gagtgagaaattcgggctcag -3’

F.Foward; R.Reverse
